# The Effects of Age, Gender, and Postvoid Residual Volume on Catheterization Rates After Treatment with OnabotulinumtoxinA for Overactive Bladder

**DOI:** 10.1016/j.euros.2023.09.013

**Published:** 2023-10-17

**Authors:** Roger Dmochowski, Christopher Chapple, Jennifer Gruenenfelder, Jun Yu, Anand Patel, Mariana Nelson, Eric Rovner

**Affiliations:** a Vanderbilt University Medical Center, Nashville, TN, USA; b Sheffield Teaching Hospitals NHS Foundation Trust, Sheffield, UK; c St. Luke’s Urology, Boise, ID, USA; d Allergan, an AbbVie Company, Sugar Land, TX, USA; e Allergan, an AbbVie Company, Marlow, UK; f Allergan, an AbbVie Company, Irvine, CA, USA; g MUSC Health Urology Services, Charleston, SC, USA

**Keywords:** Botulinum toxin type A, Clean intermittent catheterization, Demographics, Overactive bladder, Postvoid residual volume, Urinary incontinence

## Abstract

**Background:**

Transient increases in postvoid residual urine volume (PVR) requiring clean intermittent catheterization (CIC) have occurred with onabotulinumtoxinA treatment for overactive bladder (OAB).

**Objective:**

To evaluate onabotulinumtoxinA safety and the effect of age, gender, and maximum PVR (PVR_max_) on CIC initiation in adults with OAB and urinary incontinence (UI).

**Design, setting, and participants:**

This was a pooled post hoc analysis of four placebo-controlled, multicenter randomized trials that included adults with idiopathic OAB after first onabotulinumtoxinA treatment (NCT00910845, NCT00910520, NCT01767519, NCT01945489). Patients had at least three urgency UI episodes over 3 d and at least eight micturitions per day, had inadequate management with at least one anticholinergic agent, and were willing to use CIC.

**Outcome measurements and statistical analysis:**

We measured the following outcomes: PVR_max_ within 12 wk after first treatment; CIC incidence; estimated functional capacity; PVR ratio (PVR/estimated functional capacity).

**Results and limitations:**

Of 1504 patients, 87.7% were women and 88.8% were White. The mean age was 60.5 yr across 10-yr age groups, baseline PVR was 13.8–35.0 ml, and estimated functional capacity was 293.5–475.7 ml. Mean baseline PVR was 21.3 ml overall versus 34.0 ml in the group that started CIC. The CIC incidence was 6.2% for women (range 1.1–8.4%) and 10.5% for men (range 0–14.6%). Higher CIC rates were observed for PVR_max_ >350 ml (women 91.9%, men 84.6%) in comparison to PVR_max_ of 201–350 ml (women 32.5%, men 17.4%) and PVR_max_ <200 ml (women 1.2%, men 1.6%). Overall, 2/1504 patients (both women) were unable to void spontaneously. The mean PVR ratio was highest at week 2. Some subgroups had small sample sizes.

**Conclusions:**

CIC incidence was low overall, was less frequent for women, was rare with PVR_max_ ≤200 ml, and did not appear to correlate with baseline PVR.

**Patient summary:**

After onabotulinumtoxinA treatment for OAB, patients sometimes insert a catheter to help in emptying their bladder after urinating. In this study, few patients needed a catheter, especially when less urine volume remained after urination.

## Take Home Message

In this pooled analysis, most men and women receiving onabotulinumtoxinA 100 U for overactive bladder with urinary incontinence did not require clean intermittent catheterization (CIC). These findings indicate that in the absence of urinary retention, CIC may not be necessary in most patients receiving onabotulinumtoxinA.

## 1 Introduction

Overactive bladder (OAB) symptoms are reportedly more common among women (43%) than men (27%) in the USA [1] and can occur with urinary incontinence (UI), which has been associated with quality of life (QoL) impairments, anxiety, and depression [1], [2], [3]. OAB with UI is often managed initially with an anticholinergic (antimuscarinic) agent; however, many patients discontinue treatment owing to an inadequate response or systemic side effects [4], [5]. β3 adrenoreceptor agonists may be used, but patients with OAB who experience lack of efficacy may need other treatment modalities [5].

In randomized placebo-controlled trials, onabotulinumtoxinA has demonstrated significant improvements in UI, urgency, frequency, and QoL in patients with idiopathic OAB inadequately managed with an anticholinergic agent [6], [7], [8], [9]. In these trials, transient increases in postvoid residual urine volume (PVR) were reported following onabotulinumtoxinA treatment, sometimes requiring clean intermittent catheterization (CIC; ∼6.5%) [6], [7], [8], [9]. In real-world clinical practice, the possibility of greater PVR requiring catheterization may be a concern when treating OAB in patients with lower bladder contractility, who are often older [10], and may influence a physician’s decision to offer onabotulinumtoxinA treatment. However, whether patient age affects CIC incidence and outcomes following onabotulinumtoxinA treatment requires further characterization. An objective of this pooled analysis of four randomized trials was to evaluate the safety of onabotulinumtoxinA in women and men with OAB across age groups during the 12 wk after the first onabotulinumtoxinA treatment. We also assessed the impact of maximum PVR (PVR_max_) after onabotulinumtoxinA use on CIC initiation and rates of spontaneous and nonspontaneous voiding in women and men as a function of age.

## 2 Patients and methods

### 2.1 Study design

This was a pooled post hoc analysis of four randomized, double-blind, placebo-controlled trials of onabotulinumtoxinA 100 U versus placebo (NCT00910845, NCT00910520, NCT01767519, and NCT01945489) conducted in North America and Europe that have previously been described [6], [7], [8], [9]. In brief, patients were randomized 1:1 to receive onabotulinumtoxinA 100 U or matched placebo in the first treatment cycle, followed by a 12-wk open-label treatment cycle during which all patients could receive retreatment with onabotulinumtoxinA 100 U. Protocols were approved by an ethics committee or institutional review board at each site. The study conduct was aligned with Good Clinical Practice principles and all patients provided written informed consent.

### 2.2 Patient population

Adults with idiopathic OAB, at least three urgency UI episodes (UIEs) over a 3-d period, at least eight micturitions per day, and inadequate management with at least one anticholinergic medication were enrolled if they were willing to use CIC if needed. Patients were excluded if they had OAB symptoms for any known neurologic reason.

### 2.3 Assessments

PVR_max_, assessed via ultrasound or a bladder scan, was determined at any time within 12 wk after the first onabotulinumtoxinA treatment. Per protocol, CIC was initiated if urinary retention occurred, defined as PVR_max_ of ≥200 to <350 ml with relevant associated symptoms that required CIC (at the investig ator’s discretion) or if PVR_max_ was ≥350 ml regardless of symptoms (CIC required). CIC was stopped at the investigator’s discretion if PVR_max_ and/or any associated symptoms decreased. CIC incidence was assessed for each PVR_max_ category and age group, and rates of spontaneous and nonspontaneous voiding were calculated. CIC duration was also assessed. Functional bladder capacity was estimated by summing baseline PVR and baseline volume voided per micturition (ascertained from 24-h patient bladder diaries). The PVR ratio was calculated as (PVR at each time point/estimated functional capacity) × 100. The incidence of treatment-emergent adverse events (TEAEs) was monitored throughout the studies.

### 2.4 Statistical analysis

Data for patients receiving their first treatment with onabotulinumtoxinA for OAB were analyzed and summarized descriptively. Demographics and baseline characteristics were analyzed in the pooled intent-to-treat population (all randomized patients). Patients were stratified by age (<40, 40–49, 50–59, 60–69, 70–79, and ≥80 yr) and PVR_max_ (0–200, 201–350, and >350 ml). Safety was analyzed in the pooled safety population (all patients who took at least one dose of study treatment). The software program used for the analyses was SAS v9.4 (SAS Institute, Cary, NC, USA).

## 3 Results

### 3.1 Patient demographics and baseline characteristics

Among 1504 patients with OAB in the intent-to-treat population, 87.7% were women and 88.8% were White, and the mean age was 60.5 yr (Table 1). The mean OAB duration was 6.7 yr, the daily average number of UIEs was 5.4, and the daily average number of urgency UIEs was 4.9.

**Table 1:** Patient demographics and baseline characteristics in the pooled intent-to-treat population of all randomized patients

Characteristic	Overall (*N* = 1504)	Female (*n* = 1319)	Male (*n* = 185)
Age (yr)			
Mean (SD)	60.5 (13.5)	60.4 (13.4)	61.8 (14.8)
Median (range)	62 (18–90)	62 (19–90)	65 (18–88)
Age group, *n* (%)			
<40 yr	113	97 (7.4)	16 (8.6)
40–49 yr	184	162 (12.3)	22 (11.9)
50–59 yr	351	319 (24.2)	32 (17.3)
60–69 yr	455	399 (30.3)	56 (30.3)
70–79 yr	307	264 (20.0)	43 (23.2)
≥80 yr	94	78 (5.9)	16 (8.6)
Race, *n* (%)			
Asian	16	14 (1.1)	2 (1.1)
Black	60	47 (3.6)	13 (7.0)
Hispanic	35	28 (2.1)	7 (3.8)
White	1335	1179 (89.4)	156 (84.3)
Non-White	46	39 (3.0)	7 (3.8)
Other	12	12 (0.9)	0 (0.0)
OAB duration (yr)			
Patients with data available	1498	1313	185
Mean (SD)	6.7 (7.7)	7.0 (8.1)	4.9 (3.9)
Median (range)	5.0 (0.5–64.6)	5.0 (0.5–64.6)	4.8 (0.7–23.4)
Daily UIEs (*n*)			
Mean (SD)	5.4 (3.6)	5.5 (3.5)	4.9 (3.9)
Median (range)	4.7 (0.0–23.0)	4.7 (0.0–23.0)	3.7 (1.0–21.7)
Daily urgency UIEs (*n*)			
Mean (SD)	4.9 (3.3)	4.9 (3.3)	4.4 (3.6)
Median (range)	4.0 (0.0–22.7)	4.0 (0.0–22.7)	3.3 (0.3–21.7)
PVR in the overall population (ml)			
Patients with data available	1483	1301	182
Mean (SD)	21.3 (27.4)	20.8 (27.4)	25.1 (26.8)
Median (range)	10.0 (0.0–158.0)	9.0 (0.0–158.0)	16 (0.0–97.4)
PVR in the CIC population (ml)			
Patients with data available (*n*/*N*)	85/91	69/74	16/17
Mean (SD)	34.0 (36.1)	32.5 (36.2)	40.7 (35.9)
Median (range)	20.0 (0.0–153.0)	20.0 (0.0–153.0)	32.0 (0.0–95.0)

CIC = clean intermittent catheterization; OAB = overactive bladder; PVR = postvoid residual urine volume; SD = standard deviation; UIEs = urinary incontinence episodes.

Baseline characteristics for men and women were generally well balanced, with a few exceptions (Table 1). There was a higher proportion of women than men in the 50–59-yr, 70–79-yr, and ≥80-yr age groups (Table 1). The mean OAB duration, daily average number of UIEs, and daily average number of urgency UIEs were higher for women than for men (Table 1).

Baseline PVR was not notably higher for patients who started CIC after onabotulinumtoxinA treatment (34.0 ml) than for the overall population (including those who did not perform CIC; 21.3 ml).

### 3.2 CIC incidence across age groups

**Fig. 1:**
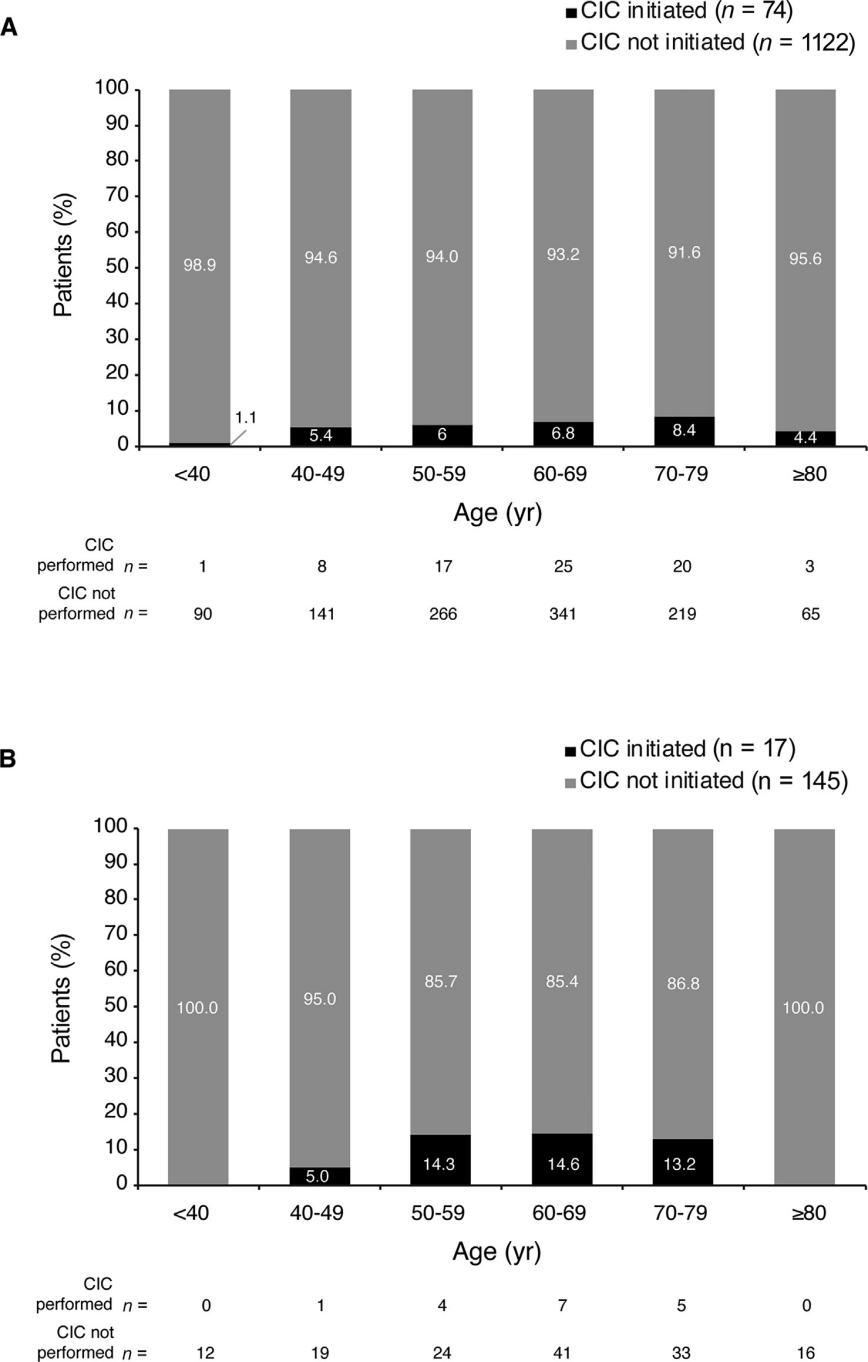
Clean intermittent catheterization (CIC) incidence for (A) female and (B) male patients, stratified by age group.

In the first 12 wk following onabotulinumtoxinA treatment, CIC incidence was low overall (6.7%, *n* = 91), low in all age groups, and lower for women (6.2%) than for men (10.5%; Fig. 1). For women, CIC incidence was highest in the 70–79-yr group (8.4%) and lowest in the <40-yr group (1.1%; Fig. 1A). For men, CIC incidence was higher in the 50–59-yr (14.3%), 60–69-yr (14.6%), and 70–79-yr (13.2%) age groups; no male patients started CIC in the <40-yr (0%) or ≥80-yr (0%) groups (Fig. 1B).

### 3.3 CIC incidence among women stratified by PVR_max_

**Fig. 2:**
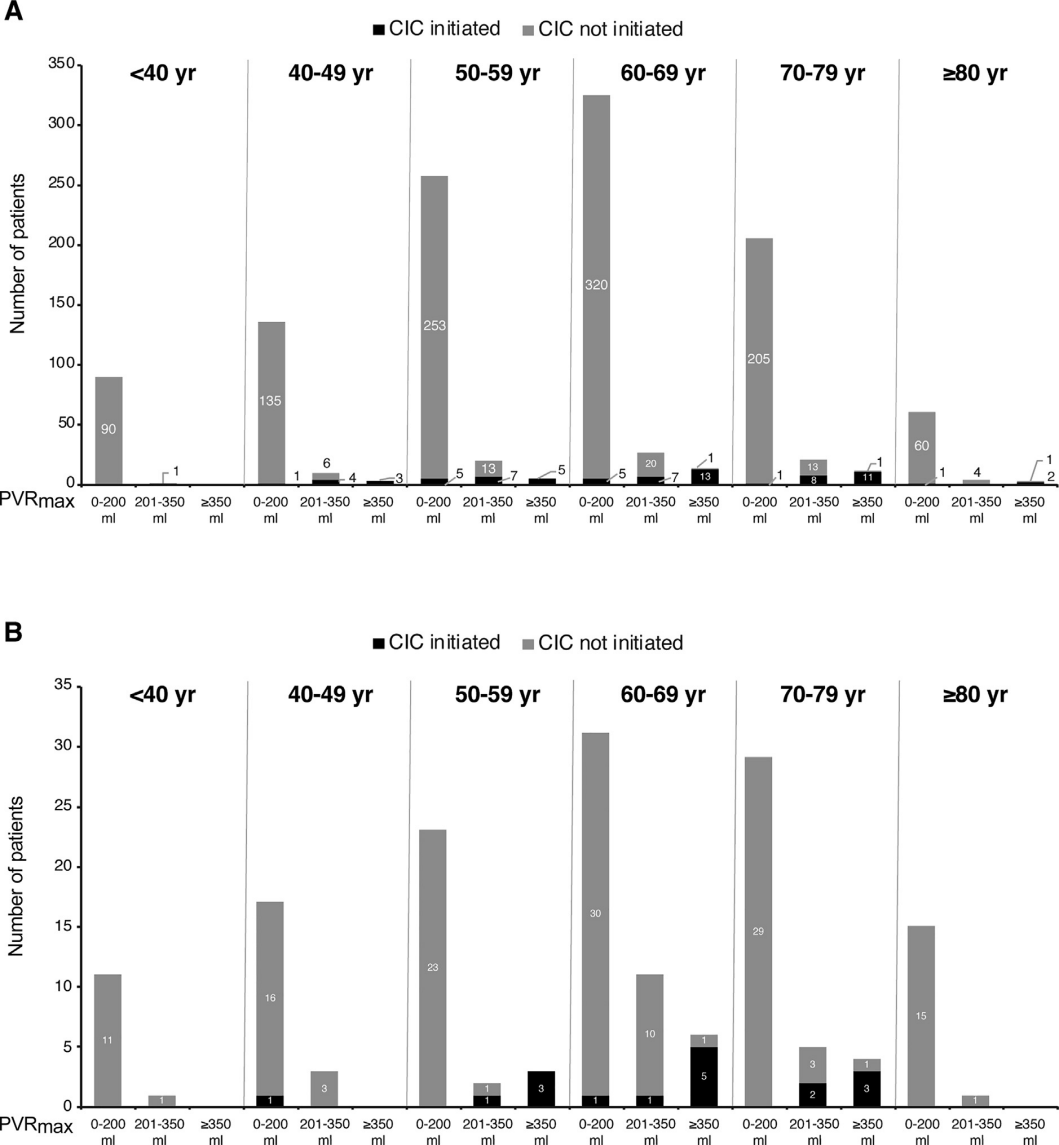
Clean intermittent catheterization (CIC) incidence stratified by age and PVR_max_ (0–200 ml, 201–350 ml, and >350 ml) for (A) female and (B) male patients. PVR_max_ = maximum postvoid residual urine volume.

Among 1196 female patients, postbaseline PVR_max_ was 0–200 ml in 1076 (90.0%), 201–350 ml in 83 (6.9%), and >350 ml in 37 (3.1%). Seventy-four women (6.2%) started CIC, among whom PVR_max_ was 0–200 ml in 13, 201–350 ml in 27, and >350 ml in 34 (Fig. 2). Few women with PVR_max_ of 0–200 ml started CIC (13/1076; 1.2%), and the number was low across all age groups (Fig. 2). Among women with PVR_max_ of 201–350 ml, 32.5% (27/83) started CIC (Fig. 2). There were three incidences of CIC among women aged ≥80 yr across PVR_max_ categories (Fig. 2). Among women with PVR_max_ >350 ml, 91.9% (34/37) started CIC; 3/29 women aged ≥60 yr did not start CIC despite having PVR_max_ >350 ml (Fig. 2).

### 3.4 CIC incidence among men stratified by PVR_max_

Among 162 male patients, postbaseline PVR_max_ was 0–200 ml in 126 (77.8%), 201–350 ml in 23 (14.1%), and >350 ml in 13 (8.0%). Seventeen (10.5%) of 162 men started CIC, among whom PVR_max_ was 0–200 ml in two, 201–350 ml in four, and >350 ml in 11 (Fig. 2). The CIV incidence among men with PVR_max_ of 0–200 ml was low overall (2/126; 1.6%) and across all age groups (Fig. 2). Among men with PVR_max_ of 201–350 ml, 17.4% (4/23) started CIC; there were no incidences of CIC in the youngest (≤49 yr) and oldest (≥80 yr) age groups (Fig. 2). Among men with PVR_max_ >350 ml, 84.6% (11/13) started CIC; 2/10 men in the group aged 60–79 yr did not start CIC despite having PVR_max_ >350 ml (Fig. 2).

### 3.5 Mean CIC duration across age and PVR_max_ groups

Among patients who needed to perform CIC and for whom CIC duration data were available, the mean CIC duration overall was 65.5 d (standard deviation [SD] 66.2) and was 63.4 d for men (SD 56.8; *n* = 13) and 65.9 d for women (SD 68.7; *n* = 56). The median CIC duration was 46 d overall (men 46 d, women 45 d). For both men and women, mean CIC duration varied across age and PVR_max_ subgroups (Supplementary Fig. 1), and was generally lowest for patients aged <40 yr and those with PVR_max_ <200 ml.

### 3.6 Incidence of spontaneous versus nonspontaneous voiding in the CIC cohort stratified by PVR_max_

Overall, 0.1% (2/1504) of patients were unable to void spontaneously; both were women who started CIC (Supplementary Fig. 2), representing 0.2% (2/1319) of the female population. One patient was 75 yr old with PVR_max_ of 250 ml; the other was 76 yr old with PVR_max_ of 700 ml. All male patients were able to void spontaneously following treatment with onabotulinumtoxinA.

### 3.7 Functional bladder capacity and PVR ratio stratified by patient characteristics

Across all age groups, baseline PVR was low. Average baseline functional bladder capacity ranged from 294 to 476 ml, with considerable variability (Table 2). The mean PVR ratio was highest at week 2, regardless of age or the need to initiate CIC, and was higher at all time points for male than for female patients (Supplementary Figs. 3–6).

**Table 2:** Baseline PVR and EFC by age category

Age	Patients	Mean baseline result (SD)	
category	(*n*)	PVR (ml)	EFC (ml)
<40 yr	105	13.8 (20.7)	293.5 (456.5)
40–49 yr	170	23.6 (28.0)	357.5 (536.5)
50–59 yr	312	17.0 (23.9)	444.7 (615.8) a
60–69 yr	413	21.4 (28.6)	475.7 (724.2)
70–79 yr	280	23.0 (28.4)	346.7 (467.3)
80–89 yr	83	22.7 (26.0)	294.3 (400.5)
≥90 yr	1	35.0 (NA)	–

EFC = estimated functional capacity (baseline PVR + baseline volume voided per micturition); NA = not applicable; PVR = postvoid residual urine volume; SD = standard deviation.

a Data missing for 1 patient.

### 3.8 Safety

Among 1239 female patients (onabotulinumtoxinA 729; placebo 510) in the safety population, 412 (56.5%) in the onabotulinumtoxinA group and 266 (52.2%) in the placebo group experienced at least one AE (Table 3). The TEAEs most commonly reported (≥3% of patients) for women were urinary tract infection, bacteriuria, dysuria, and urine retention. Among 168 male patients (onabotulinumtoxinA 96; placebo 72), 46 (47.9%) in the onabotulinumtoxinA group and 33 (45.8%) in the placebo group experienced at least one AE (Table 3). The TEAEs most commonly reported (≥3% of patients) for men were urinary tract infection, residual urine volume, dysuria, hematuria, pollakiuria, and urinary retention (Table 3).

**Table 3:** Safety and tolerability in the pooled safety population for patients with data available at week 12

	Patients, *n* (%)		
	Placebo	OA	All
**Female patients**	510	729	1239
Patients with any AE	266 (52.2)	412 (56.5)	678 (54.7)
TEAEs in ≥3% patients			
Bacteriuria	17 (3.3)	30 (4.1)	47 (3.8)
Urinary tract infection a	103 (20.2)	152 (20.9)	255 (20.6)
Dysuria	24 (4.7)	55 (7.5)	79 (6.4)
Urinary retention b	22 (4.3)	41 (5.6)	63 (5.1)
**Male patients**	72	96	168
Patients with any AE	33 (45.8)	46 (47.9)	79 (47.0)
TEAEs in ≥3% patients			
Urinary tract infection a	4 (5.6)	8 (8.3)	12 (7.1)
Residual urine volume	5 (6.9)	8 (8.3)	13 (7.7)
Dysuria	6 (8.3)	7 (7.3)	13 (7.7)
Hematuria	4 (5.6)	7 (7.3)	11 (6.5)
Pollakiuria	2 (2.8)	4 (4.2)	6 (3.6)
Urinary retention b	9 (12.5)	8 (8.3)	17 (10.1)

AE = adverse event; CIC = clean intermittent catheterization; OA = onabotulinumtoxinA 100 U; PVR = postvoid residual urine volume; TEAE = treatment-emergent AE.

a Urinary tract infection was defined as a positive urine culture result with a bacteriuria count of >10^5^ cfu/ml and leukocyturia of >5 cells per high-power field, regardless of the patient’s symptoms.

b Urinary retention was defined as PVR ≥200 ml requiring CIC. CIC was initiated if PVR was ≥350 ml regardless of symptoms, or ≥200 to <350 ml with associated symptoms that, in the investigator’s opinion, required CIC.

## 4 Discussion

In this pooled analysis of four randomized clinical trials of onabotulinumtoxinA 100 U for the treatment of OAB with UI [6], [7], [8], [9], the incidence of CIC was low overall, particularly for women and younger patients, and especially for those with PVR_max_ ≤200 ml, consistent with prior findings [11]. There was no apparent relationship between baseline PVR and CIC initiation, and few patients met the clinical criterion for urinary retention, with only 0.1% of patients unable to spontaneously void following onabotulinumtoxinA treatment. These findings support past conclusions that CIC may not be necessary in most patients treated with onabotulinumtoxinA, as clinical urinary retention is rare and most patients did not develop significantly elevated PVR. These data provide additional context for clinicians regarding elevated PVR following onabotulinumtoxinA treatment.

CIC rates were low, especially in the younger age groups (<50 yr), with the highest rates observed in the 70–79-yr group for women and the 50–79-yr group for men. These findings are consistent with research showing significant increases in PVR and decreases in contractility after the age of 50 yr in women [12], suggesting that the risk of needing CIC after treatment increases with age. In this study, CIC was used primarily by patients with PVR_max_ ≥350 ml according to the study protocols [6], [7], [8], [9].

Most patients receiving onabotulinumtoxinA could void spontaneously, and very few met the International Continence Society (ICS) clinical criterion for urinary retention (inability to pass urine despite persistent effort) [13]. Complete inability to void was rare and observed in only 2/1319 women, both of whom were aged >70 yr with PVR_max_ >200 ml; none of the men were unable to spontaneously void after onabotulinumtoxinA treatment.

In both males and females, the PVR ratio after onabotulinumtoxinA was highest at week 2 and declined thereafter. This observation is consistent with the change in PVR observed over time in the current analysis and coincides with the onset of treatment efficacy in clinical trials [6], [7], [9]. OnabotulinumtoxinA 100 U was well tolerated in women and men in all age groups, with no unexpected safety signals. The TEAE profile was consistent with previous publications and generally limited to local urologic events.

The CIC incidence observed in the current analysis (women 6%, men 10%) is higher than in recent real-world reports (ranging from 1.6% to 2.7%) [14], [15]. This discrepancy between clinical trials and real-world studies probably reflects the close monitoring and specific criteria for initiating CIC use in the clinical trials and suggests that a different standard for CIC initiation is being applied in clinical practice. Furthermore, changes in real-world practice since the completion of these trials may have contributed to differences in CIC rates.

When deciding whether to initiate CIC, PVR should be interpreted in the context of other clinical characteristics. For example, in patients with PVR_max_ in the range 201–350 ml, functional bladder capacity and symptoms should be considered, as some patients with large functional bladder capacity may remain asymptomatic despite elevated PVR, and some asymptomatic patients may not require CIC [16], [17]. It is important also to consider that functional bladder capacity can vary, for example, according to behavioral stressors and external stimulants [18], [19].

In this analysis, a few patients with a PVR_max_ ≥350 ml, including one older patient (≥80 yr), did not start CIC. In practice, the impact of factors such as low vision and dexterity issues on CIC initiation should be considered in the older patient population. Although younger patients may be more able to perform CIC, evaluation of their functional bladder capacity and PVR may help in establishing if CIC is actually necessary.

This analysis provides valuable data for men, who are often under-represented in the literature on OAB. The proportion of male patients starting CIC was low across the age groups, particularly in the group with PVR_max_ <200 ml, and no male patients aged <40 yr or ≥80 yr started CIC. However, the small male population in this study limits the interpretability of these findings.

This pooled analysis was conducted post hoc. The PVR_max_ categories are somewhat arbitrary and may not be aligned with any specific clinical practice. Few patients started CIC in some age and PVR_max_ groups, limiting the interpretability of the associated findings, and caution is advised when interpreting CIC duration by age and sex owing to small sample sizes in certain groups. PVR_max_ ranges used in these analyses may not be relevant for all patients, as functional capacity varies among individuals. The estimated functional capacity was calculated as a proxy given the limitations of the data available, and this analysis did not consider exogenous factors that may have affected this parameter.

Future studies evaluating the influence of patient characteristics such as diabetes mellitus or other comorbidities on CIC incidence may build on this work in furthering our understanding of any increase in the risk of PVR elevation after treatment in specific populations [20]. In addition, multivariable analysis of age, baseline PVR, and minimum/maximum PVR (a measure of best and worst contractility) as independent variables may provide insight into the combined role of age and PVR in predicting the need for CIC.

## 5 Conclusions

In this pooled analysis of onabotulinumtoxinA 100 U treatment for OAB with UI, mean PVR at baseline increased with age and was similar for the overall population and for patients who started CIC. Most women and men did not develop markedly raised PVR necessitating CIC. The mean PVR ratio was highest at week 2, inability to spontaneously void after onabotulinumtoxinA treatment was extremely rare, and few patients had urinary retention according to the ICS criteria. These data on CIC incidence after onabotulinumtoxinA treatment according to patient characteristics may facilitate more individually tailored counseling on the risk of developing elevated PVR requiring CIC.

These data were presented in part at ICS 2022, Vienna, Austria and TOXINS 2022, New Orleans, LA, USA.

  ***Author contributions***: Eric Rovner had full access to all the data in the study and takes responsibility for the integrity of the data and the accuracy of the data analysis.

  *Study concept and design*: Rovner, Chapple.

*Acquisition of data*: Chapple.

*Analysis and interpretation of data*: All authors.

*Drafting of the manuscript*: Rovner, Chapple.

*Critical revision of the manuscript for important intellectual content*: All authors.

*Statistical analysis*: Yu.

*Obtaining funding*: Rovner, Chapple.

*Administrative, technical, or material support*: None.

*Supervision*: None.

*Other*: None.

  ***Financial disclosures*:** Eric Rovner certifies that all conflicts of interest, including specific financial interests and relationships and affiliations relevant to the subject matter or materials discussed in the manuscript (eg, employment/affiliation, grants or funding, consultancies, honoraria, stock ownership or options, expert testimony, royalties, or patents filed, received, or pending), are the following: Roger Dmochowski has served as an advisor for AbbVie. Christopher Chapple has served on an advisory board and as a researcher for Allergan; has served as an author for Astellas, B Braun, and Ferring, as a speaker for Allergan, Astellas, and Urovant, and as a consultant for Proverum and Ferring; and is joint holder of a patent for a new mesh material in development. Jennifer Gruenenfelder has served on a speaker bureau for AbbVie. Jun Yu, Anand Patel, and Mariana Nelson are employees of AbbVie and may hold AbbVie stock. Eric Rovner has served on an advisory board for Allergan/AbbVie and as a study participant and central urodynamics reader for prior Allergan onabotulinumtoxinA studies.

  ***Funding/Support and role of the sponsor*:** Allergan (now AbbVie) funded this study and contributed to the study design, collection, analysis, and interpretation of the data, and review and approval of the manuscript for publication. All authors had access to relevant data and participated in the drafting, review, and approval of this publication. No honoraria or payments were made for authorship.

  ***Acknowledgments*:** Medical writing support was provided by Illyce Nuňez (Peloton Advantage, LLC, an OPEN Health company, Parsippany, NJ, USA) and was funded by AbbVie.

  ***Data sharing statement:*** AbbVie is committed to responsible data sharing regarding the clinical trials the company sponsors. This includes access to anonymized, individual, and trial-level data (analysis data sets), as well as other information (eg, protocols, clinical study reports, and analysis plans), as long as the trials are not part of an ongoing or planned regulatory submission. This includes requests for clinical trial data for unlicensed products and indications. These clinical trial data can be requested by any qualified researchers who engage in rigorous, independent, scientific research, and will be provided following review and approval of a research proposal, statistical analysis plan, and execution of a data sharing agreement. Data requests can be submitted at any time after approval in the USA and Europe and after acceptance of this manuscript for publication. The data will be accessible for 12 months, with possible extensions considered. For more information on the process or to submit a request, visit the following link: https://vivli.org/ourmember/abbvie/, then select “Home”.
