# Supplementary material for: The Effects of Age, Gender, and Postvoid Residual Volume on Catheterization Rates After Treatment with OnabotulinumtoxinA for Overactive Bladder
Source: Eur Urol Open Sci. 2023 Oct 17;57:98–105. doi: 10.1016/j.euros.2023.09.013 (PMC10658411; doi:10.1016/j.euros.2023.09.013)
Supplement: Supplementary data 1 [file mmc1.docx]

**Supplementary Figures**

**Figure S1. Mean CIC duration in female (A) and male (B) patients who initiated CIC, stratified by age group and MaxPVR volume. Dots represent individual patient data. CIC = clean intermittent catheterization; MaxPVR = maximum postvoid residual.**

**Figure S2. Rates of spontaneous and nonspontaneous voiding in female patients who initiated CIC, stratified by age group and MaxPVR volume. Dots represent individual patient data. CIC = clean intermittent catheterization; MaxPVR = maximum postvoid residual.**

**Figure S3. PVR ratio during the first 12 weeks of first onabotulinumtoxinA treatment. PVR ratio = (PVR volume at each time point/estimated functional capacity) × 100; PVR, postvoid residual; estimated functional capacity = baseline PVR volume + baseline volume voided per micturition. PVR = postvoid residual.**

**Figure S4. PVR ratio during the first 12 weeks of first onabotulinumtoxinA treatment, stratified by gender, (A) female and (B) male. PVR ratio = (PVR volume at each time point/estimated functional capacity) × 100; PVR, postvoid residual; estimated functional capacity = baseline PVR volume + baseline volume voided per micturition.** **PVR = postvoid residual.**

**Figure S5. PVR ratio during the first 12 weeks of first onabotulinumtoxinA treatment, stratified by age. PVR ratio = (PVR volume at each time point/estimated functional capacity) × 100; estimated functional capacity = baseline PVR volume + baseline volume voided per micturition. BL, baseline; PVR, postvoid residual.** **PVR = postvoid residual.**

**Figure S6. PVR ratio during the first 12 weeks of first onabotulinumtoxinA treatment, stratified by need to perform CIC. PVR ratio = (PVR volume at each time point/estimated functional capacity) × 100; estimated functional capacity = baseline PVR + baseline volume voided per micturition. CIC = clean intermittent catheterization; PVR = postvoid residual.**
